# Tendon Fascicle-Inspired Nanofibrous Scaffold of Polylactic acid/Collagen with Enhanced 3D-Structure and Biomechanical Properties

**DOI:** 10.1038/s41598-018-35536-8

**Published:** 2018-11-21

**Authors:** Alberto Sensini, Chiara Gualandi, Andrea Zucchelli, Liam A. Boyle, Alexander P. Kao, Gwendolen C. Reilly, Gianluca Tozzi, Luca Cristofolini, Maria Letizia Focarete

**Affiliations:** 1 Department of Industrial Engineering, Alma Mater Studiorum - Università di Bologna, 40131 Bologna, Italy; 2 Department of Chemistry “G. Ciamician” and National Consortium of Materials Science and Technology (INSTM, Bologna RU), Alma Mater Studiorum - Università di Bologna, 40126 Bologna, Italy; 3 Health Sciences and Technologies – Interdepartmental Center for Industrial Research (HST-ICIR), Alma Mater Studiorum - Università di Bologna, 40064 Ozzano dell’Emilia Bologna, Italy; 4 INSIGNEO Institute for in silico Medicine, Department of Materials Science, University of Sheffield, Sheffield, S10 2TN United Kingdom; 5 ZEISS Global Centre, School of Mechanical and Design Engineering, University of Portsmouth, PO1 3DJ, Portsmouth, UK

## Abstract

Surgical treatment of tendon lesions still yields unsatisfactory clinical outcomes. The use of bioresorbable scaffolds represents a way forward to improve tissue repair. Scaffolds for tendon reconstruction should have a structure mimicking that of the natural tendon, while providing adequate mechanical strength and stiffness. In this paper, electrospun nanofibers of two crosslinked PLLA/Collagen blends (PLLA/Coll-75/25, PLLA/Coll-50/50) were developed and then wrapped in bundles, where the nanofibers are predominantly aligned along the bundles. Bundle morphology was assessed via SEM and high-resolution x-ray computed tomography (XCT). The 0.4-micron resolution in XCT demonstrated a biomimetic morphology of the bundles for all compositions, with a predominant nanofiber alignment and some scatter (50–60% were within 12° from the axis of the bundle), similar to the tendon microstructure. Human fibroblasts seeded on the bundles had increased metabolic activity from day 7 to day 21 of culture. The stiffness, strength and toughness of the bundles are comparable to tendon fascicles, both in the as-spun condition and after crosslinking, with moderate loss of mechanical properties after ageing in PBS (7 and 14 days). PLLA/Coll-75/25 has more desirable mechanical properties such as stiffness and ductility, compared to the PLLA/Coll-50/50. This study confirms the potential to bioengineer tendon fascicles with enhanced 3D structure and biomechanical properties.

## Introduction

Ruptures and lesions of tendons are very common in elderly people, but also in athletes and young adults, deriving from chronic tendinopathies, acute injuries due to inflammatory processes, or trauma^1–3^. Frequently injured tendons are, for example, the shoulder rotator cuff, the flexor, the Achilles and the patellar^4,5^. Among others, Achilles tendon rupture is a common sports-related injury, with the highest incidence observed in 30- to 50-years old males, that often results in disability with degeneration occurring in an estimated 11% of runners^6–8^. Surgical treatment is the standard therapy for the majority of patients and includes minimally invasive, percutaneous or open repair strategies, depending on the extent of the injury. Unfortunately, postoperative complications often occur, with associated re-rupture risk: for example the Achilles tendon re-fracture occurs in 8–13% cases and for the flexor/extensor tendon in 4–18% cases^7,9,10^. Furthermore, the formation of scar tissue generates morphological discontinuities, which impair the mechanical properties and the proper biomechanical functionality of the tendon^11^. In order to avoid this complication, often surgeons tailor the use of autografts, allografts, xenografts, or tendon prostheses and/or sutures, depending on the site and severity of the injury^12^. Autologous grafts are immunologically suitable, but are often associated with some degree of donor morbidity, whereas allografts are not widely available, can be expensive and carry the risk of rejection and transmission of disease. Implants made of inert synthetic materials, typically made from non-resorbable polymers such as polytetrafluoroethylene (PTFE), polythiophene (PTP), polyethylene (PE) or silicone, are relatively successful in reconstructive surgery since they initially have good postoperative mechanical properties. However, inert synthetic implants have poor long-term effectiveness as their mechanical properties degrade over time due to wear, while the residual tendon tissue can be compromised due to stress shielding^2,3,13–15^. Other drawbacks with artificial tendon prostheses are inflammatory responses, failure at the fixation sites, and lack of long-term biocompatibility^13–16^. For these reasons, a tissue engineering (TE) approach represents a promising solution for tendon reconstruction, prompted also by the increasing development of biocompatible and resorbable scaffolds. The primary role of scaffolds in tendon TE is to provide temporary structural and mechanical support to promote tissue healing. Scaffolds can uptake the loads during the initial phase of repair of the injured tendon. By accurately tuning the rate of bioresorption with respect to the time needed for native tissue formation, they aim to encourage regeneration through tissue remodeling^2^. Among the various techniques to produce scaffolds for tendon tissue regeneration, electrospinning is one of the most versatile. Thanks to its ability to produce filaments of both natural and synthetic polymers with nano- and micrometric diameters oriented in specific directions, electrospinning enables the production of scaffolds morphologically similar to the hierarchical structure of the tendon collagen fascicles and fibrils^17–20^. By wrapping an electrospun mat of aligned fibers, or by mechanically rolling groups of fibers, it is possible to produce electrospun units, called bundles, composed of aligned nanofibers that resemble tendon fascicles^17,21,22^.

These scaffolds can be pre-seeded with tendon derived fibroblasts, commonly referred to as tenocytes, dermal derived fibroblasts or even stem cells. Dermal fibroblasts may be beneficial as a seeding cell compared to stem cells, as they are not able to differentiate into bone or cartilage cell lineages, which can lead to ectopic bone or cartilage formation^23^. Dermal fibroblasts also have similar characteristics to tendon derived fibroblasts and have the added benefit that they can be harvested from a simple skin biopsy^24^. Alternatively, scaffolds can be implanted directly and allow the hosts cells to migrate into and populate the scaffold.

Published literature confirms fibroblasts can proliferate on electrospun scaffolds made of resorbable materials, and their attachment and growth can be guided by the direction of fibers, both for tendon and ligament applications^17,21,25–35^. It is also well established that a combination of resorbable synthetic polymers such as poly(L-lactic acid) (PLLA), poly(lactic-co-glycolic acid) (PLGA), poly(ε-caprolactone) and natural polymers such as collagen, silk, chitosan or gelatin, are able to increase the biocompatibility and cell adhesion to these scaffolds^2,17,25,32,35^. Among the various combinations of synthetic and natural polymers proposed for producing electrospun fibers mats for tendon TE, the system composed of PLLA and collagen (Coll) represents a promising choice, since it combines the good mechanical and processing properties of a synthetic component with the bioactivity of a natural polymer^17,35^. Some groups have investigated blends of PLLA and Coll^17,35–39^. The two polymers have also been electrospun as separate phases by means of a coaxial electrospinning process^40^. The two main challenges that scaffolds for tendon TE face at present are: (i) providing adequate mechanical strength to meet the *in vivo* requirements, with a stiffness matching that of the natural tendon, and (ii) having a 3D structure and architecture that closely resemble the complex multiscale organization of native tendon tissue. An important aspect in the development of scaffolds for tendon TE, in addition to assessing their biomechanical properties, is accurately evaluating the 3D structure and morphology.

**Figure 1:**
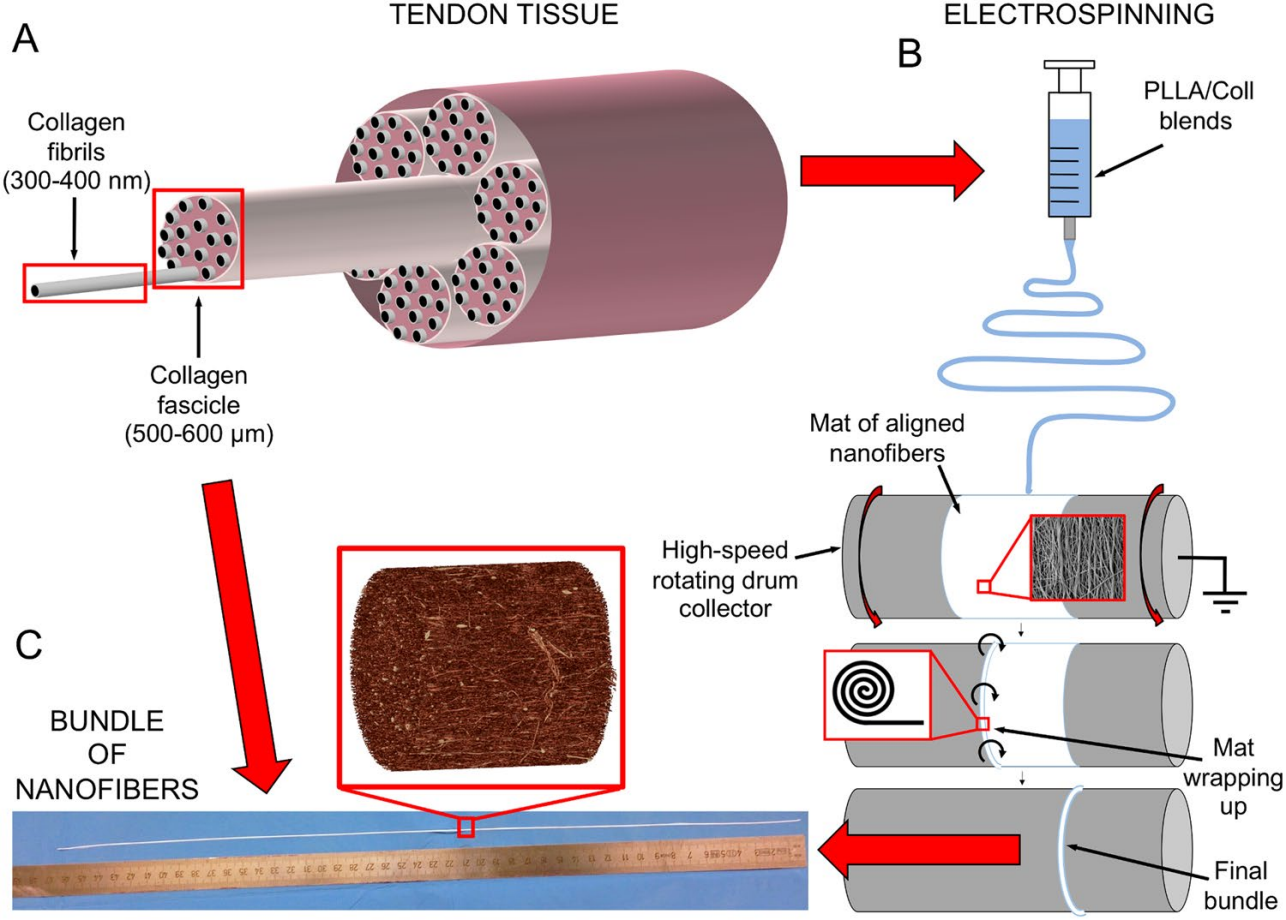
Sketch of tendon structure highlighting the three main hierarchical levels of aggregation (nomenclature derived from Kastelic *et al*.^18^) (**A**), schematic of the fabrication process (**B**) and photograph of the final bundle resembling the structure of tendon fascicle (**C**). The individual electrospun nanofibers mimic the natural collagen fibrils. The bundle containing a number of nanofibers mimics the fascicles of collagen in the natural tendon.

In the present work, crosslinked PLLA/Coll electrospun bundles were developed, with a 3D structure suitable to mimic the tendon hierarchical structure (Fig. 1A), and with enhanced mechanical properties, in the range of human tendon fascicles. The biomechanical properties of the scaffolds were evaluated in detail, and *in vitro* tests were performed to assess cell adhesion and metabolic activity. High-resolution x-ray computed tomography (XCT) was also used to undertake a detailed evaluation of the bundle internal morphology and the effects of the crosslinking process on nanofiber morphology, distribution and alignment.

## Results

### Morphology of Nanofibers and Bundles

Electrospun bundles of the two compositions PLLA/Coll-75/25 and PLLA/Coll-50/50 were produced as previously reported, by means of a high-speed rotating drum collector (Fig. S1) that allowed the production of nanofibers preferentially aligned in the direction of the drum rotation^17^. By rolling up the electrospun mat along the axis of the drum (Fig. 1B), bundles of several centimeters in length were obtained (Fig. 1C). The individual electrospun nanofibers mimicked the natural collagen fibrils. The bundle containing a number of nanofibers mimicked the fascicles of collagen in the natural tendon.

**Figure 2:**
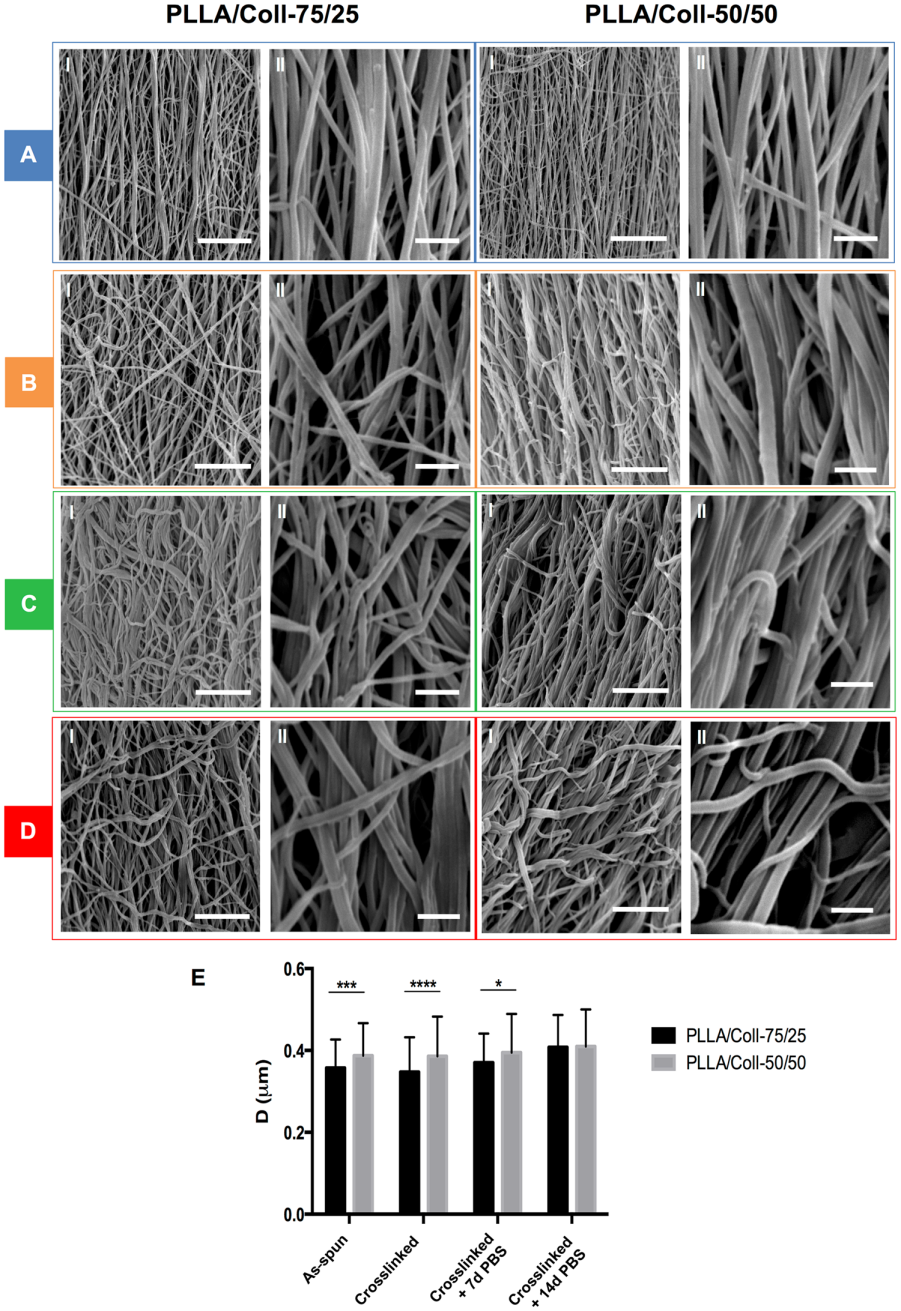
SEM images of PLLA/Coll-75/25 and PLLA/Coll-50/50 as-spun (**A**), immediately after cross-linking (**B**), after 7 days in PBS (**C**) and after 14 days in PBS (**D**). For each case, images at two different magnifications are presented (Scale bars: I = 10 μm; II = 2 μm). The mean and standard deviation of nanofiber diameters (**E**) is plotted for the two compositions together with statistical significance of post-hoc comparisons (Tukey multiple comparisons, *P ≤ 0.05, **P ≤ 0.01, ***P ≤ 0.001, ****P ≤ 0.0001).

Bundles consisted of bead-free nanometric fibers (Fig. 2). Alteration of nanofiber morphology, as a consequence of the crosslinking treatment (which was performed as described in the Experimental Section to delay collagen loss), and the ageing process in phosphate buffered saline (PBS) for different time intervals, were assessed by SEM observations (Fig. 2). As-spun PLLA/Coll-75/25 and PLLA/Coll-50/50 nanofibers had similar diameters of 0.36 ± 0.07 μm and 0.39 ± 0.09 μm (mean ± standard deviation) respectively (Fig. 2E). Nanofibers retained the same morphology and similar diameter after the crosslinking treatment and after 7 days of immersion in PBS, while both compositions showed a slight swelling of the nanofibers leading to higher nanofiber diameters after 14 days (Fig. 2: this difference, even if relatively small, was statistically significant due to the large sample size). Moreover, after PBS immersion there were no visible cracks, welds or loss of material (Fig. 2B–D). However, after crosslinking the nanofibers assumed a slightly wavy appearance, especially in the case of the PLLA/Coll-50/50 blend.

The PLLA/Coll-75/25 bundles as-spun had a diameter of 624.9 ± 38.8 μm; the PLLA/Coll-50/50 bundles as-spun had a diameter of 643.1 ± 29.2 μm. Bundles as-spun of both compositions appeared flexible in handling in the dry state. After crosslinking the PLLA/Coll-75/25 and PLLA/Coll-50/50 bundles had a diameter of 575.1 ± 43.9 μm and 433.8 ± 37.3 μm, respectively. After crosslinking, the dry bundles were more brittle to handle.

The bundle did not have a measurable change in diameter after 7 and 14 days immersion in PBS, but became more brittle in handling. However, when re-immersed in PBS, all bundles regained flexibility. It is worth mentioning that immediately after crosslinking both PLLA/Coll-75/25 and PLLA/Coll-50/50 bundles shrunk in length with a decrease of about 21.5 ± 1.1% of the original length, as a consequence of PLLA chain relaxation occurring when macromolecules in the amorphous state acquire mobility^41^.

**Figure 3:**
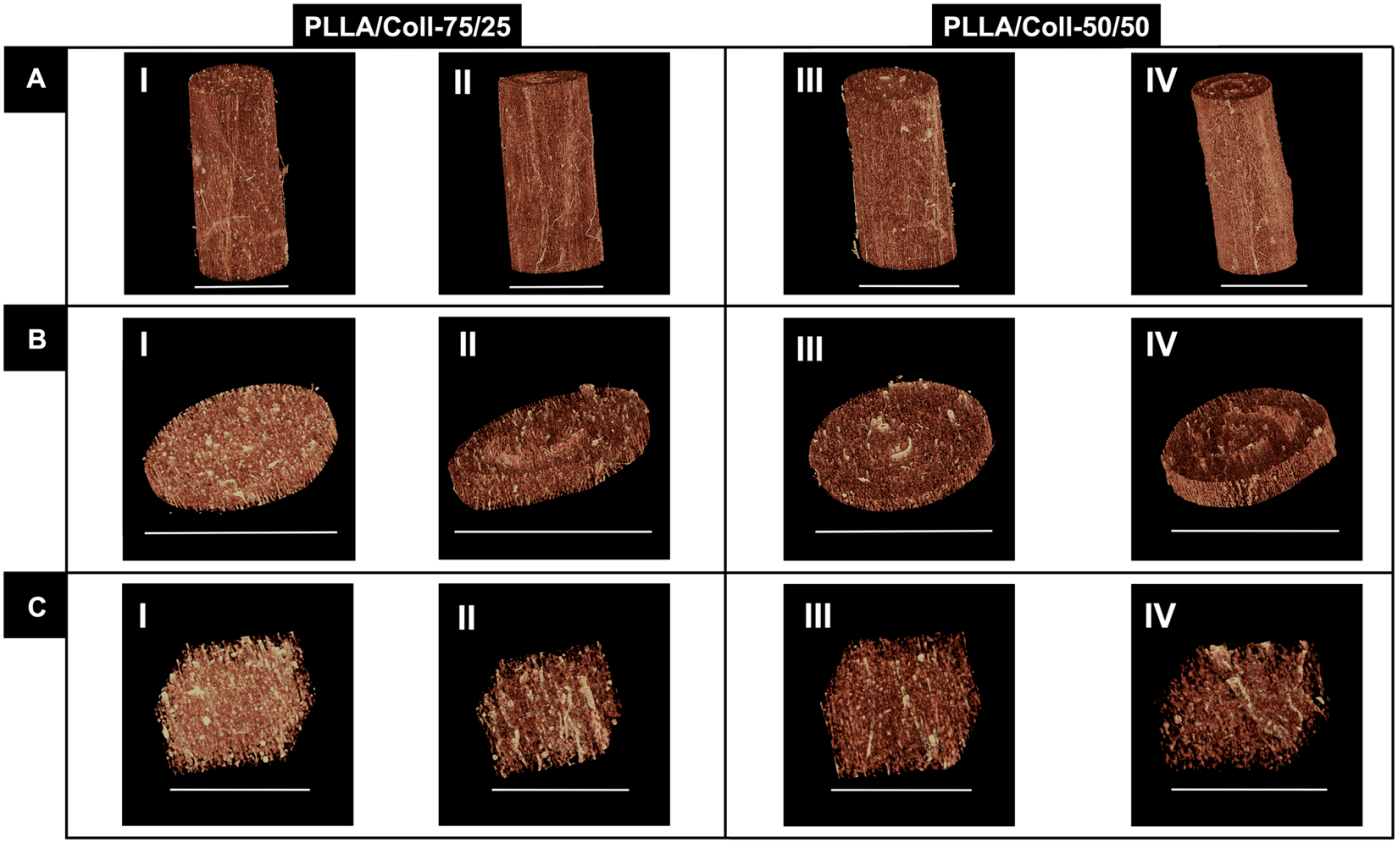
High-resolution x-ray computed tomography (XCT) images of the bundles at 1 μm voxel size: bundle segment (**A**), slice of a bundle (**B**) and magnification of cropped internal sub-volume (**C**) (**A** and **B** scale bar = 500 μm, **C** scale bar = 200 μm). PLLA/Coll-75/25 as-spun (I) and after crosslinking (II). PLLA/Coll-50/50 as-spun (III) and after crosslinking (IV).

**Figure 4:**
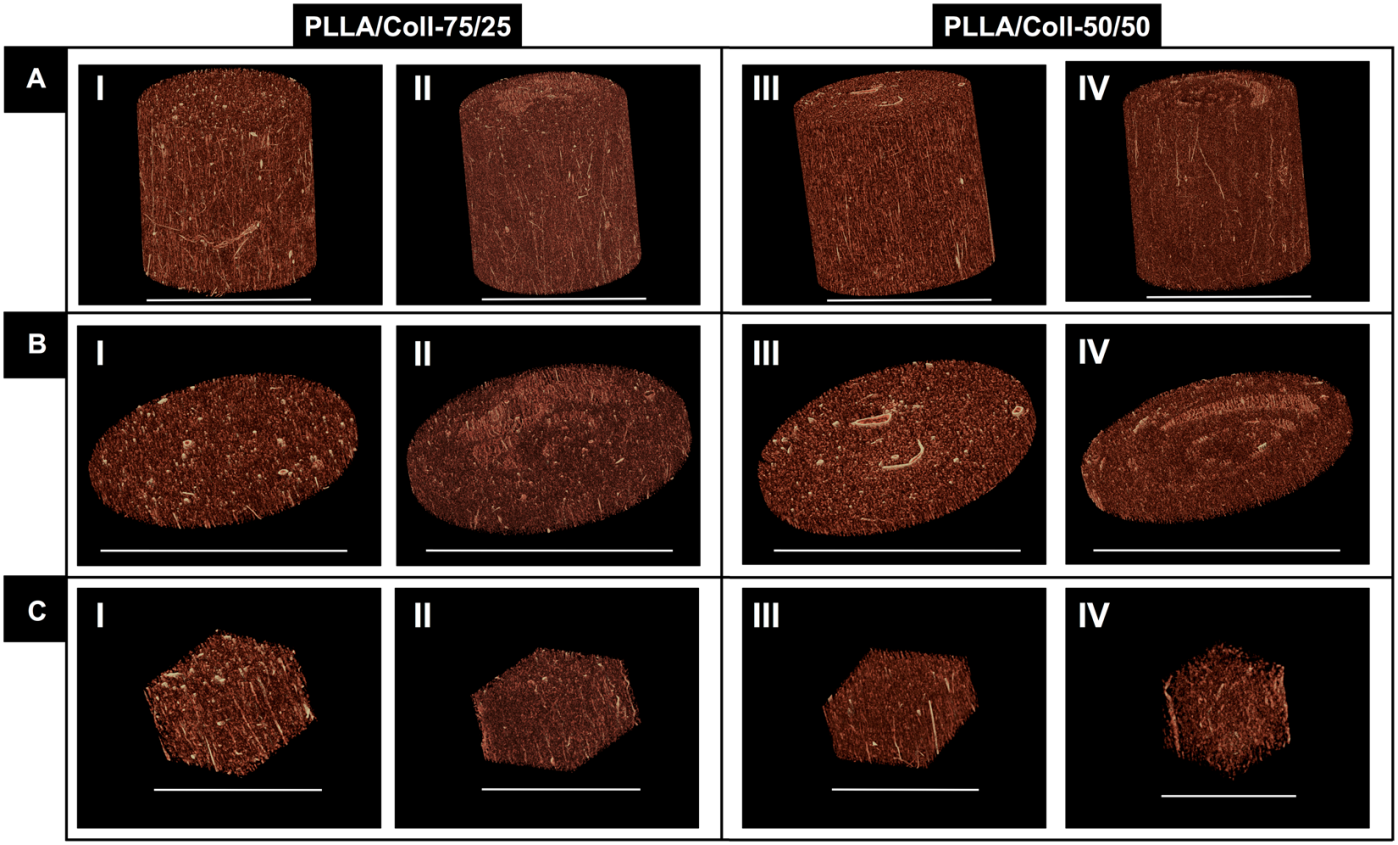
High-resolution x-ray computed tomography (XCT) images of the bundles at 0.4 μm voxel size: bundle segment (**A**), slice of a bundle (**B**) and magnification of cropped internal sub-volume (**C**) (**A** and **B** scale bar = 500 μm, **C** scale bar = 200 μm). PLLA/Coll-75/25 as-spun (I) and after crosslinking (II). PLLA/Coll-50/50 as-spun (III) and after crosslinking (IV).

High-resolution x-ray computed tomography (XCT) investigation with 1 μm (Fig. 3) and 0.4 μm (Fig. 4 and Movies in Supporting Information for as-spun and crosslinked PLLA/Coll-75/25) voxel sizes, confirmed the aligned morphology of the nanofibers for the PLLA/Coll-75/25 and PLLA/Coll-50/50 blends both for the as-spun and crosslinked bundles. Scans with 1 μm voxel size did not enable clear discernment of the nanofibers (Fig. 3). However, at 0.4 μm voxel size the nanofibers were clearly visible and very well defined (Fig. 4). Negligible loss of material was observed in the XCT images within the crosslinked bundles.

**Figure 5:**
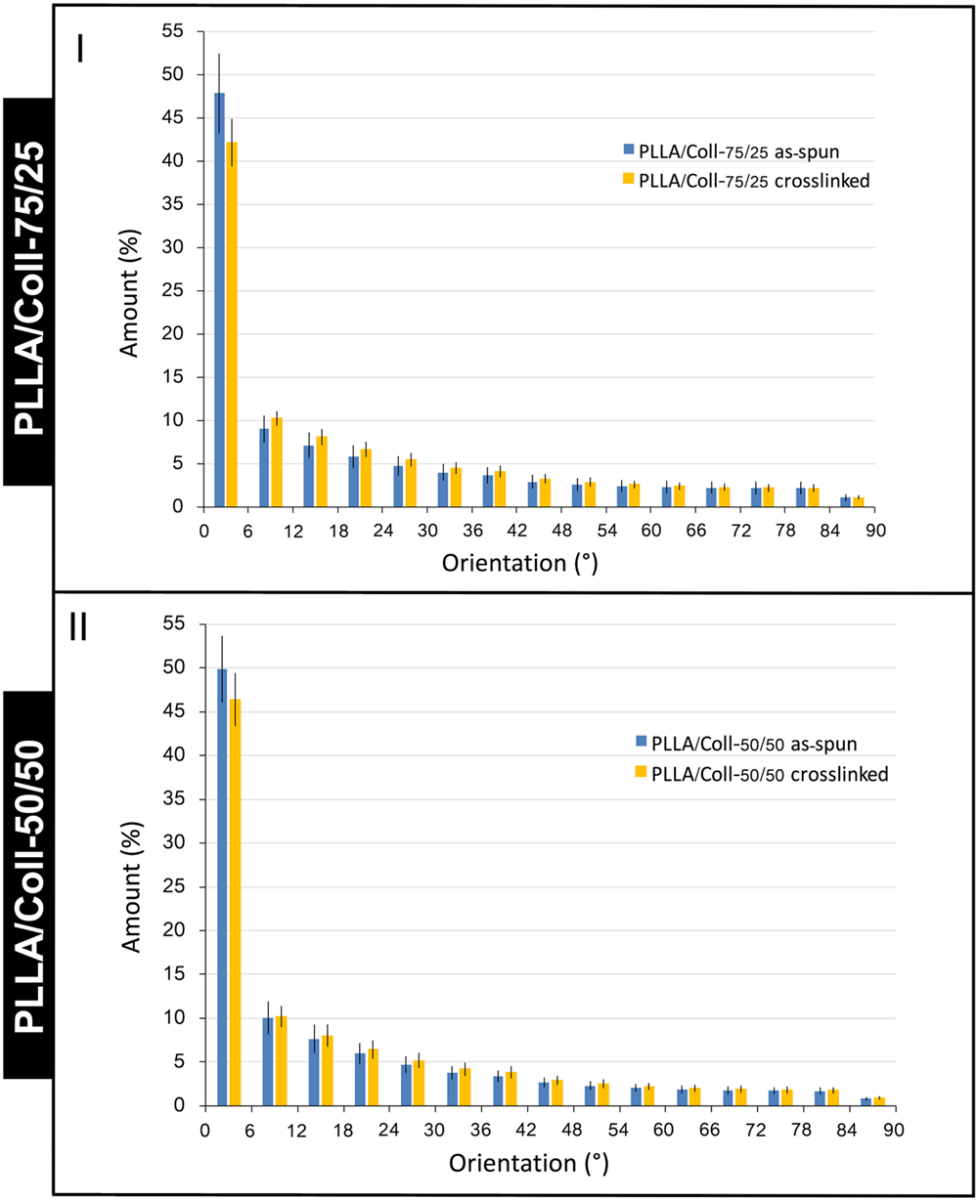
Orientation of the nanofibers measured in the XCT scans at 0.4 μm voxel size for the PLLA/Coll-75/25 (I) and PLLA/Coll-50/50 (II) bundles. The histograms report the percentage of nanofibers aligned within a specific orientation from the longitudinal direction. An orientation of the nanofibers parallel to the axis of the bundle corresponds to 0°. An orientation of the nanofibers in a transverse plane corresponds to 90°. For each orientation, the mean and standard deviation are reported among all the axial slices that were obtained reslicing each scan.

The uniaxial alignment of the nanofibers, both on the surface and in the body of the samples, as well as the retention of morphology after crosslinking and PBS immersion, were confirmed by the “Directionality analysis” on the 0.4 μm voxel size scans (Fig. 5).

The XCT morphological analysis showed that the PLLA/Coll-75/25 bundles as-spun had 56.9 ± 3.1% of the nanofibers aligned within ±12° from the longitudinal axis of the bundle, whereas the PLLA/Coll-75/25 crosslinked had 52.4 ± 1.8% of the nanofibers aligned within ±12° from the axis of the bundle (Fig. 5I), with a mean reduction of alignment of 4.5%. The PLLA/Coll-50/50 as-spun had 59.9 ± 2.8% of the nanofibers aligned within ±12° from the axis of the bundle, whereas the PLLA/Coll-50/50 crosslinked had 56.6 ± 2.1% of the nanofibers aligned within ±12° from the axis of the bundle (Fig. 5II), with a mean reduction of alignment of 3.3%.

### Bundle Chemical Composition

TGA analysis was carried out on the bundles as-spun to verify their composition (Fig. S3). To this aim, the residual weight of the bundle at the end of the analysis was compared to that of the pure blend components (PLLA and Coll) and the actual composition of the bundles was determined by applying Equation 1. The results showed that the chemical composition of the bundles as-spun was close to the nominal one (Table 1). After crosslinking, the collagen content slightly decreased from 25 to 19 wt% in the PLLA/Coll-75/25 bundles, and from 49 to 45 wt% in the PLLA/Coll-50/50. In other words, PLLA/Coll-75/25 lost about 30% of their collagen, while PLLA/Coll-50/50 only 15%. When the crosslinked bundles were maintained in PBS at 37 °C for 7 and 14 days, the chemical composition showed a very low reduction in collagen content.

**Table 1:** Chemical composition of the bundles as-spun, after crosslinking, and after ageing in PBS at 37 °C for different times.

	Sample	PLLA:Coll [wt:wt]
PLLA/Coll-75/25	As-spun	75:25^a^
	Crosslinked	(80.9 ± 0.8):(19.1 ± 0.8)^b^
	Crosslinked + 7d PBS	(80.6 ± 0.6):(19.4 ± 0.6)^b^
	Crosslinked + 14d PBS	(81.4 ± 0.1):(18.6 ± 0.1)^b^
PLLA/Coll-50/50	As-spun	51:49^a^
	Crosslinked	(54.6 ± 0.1):(45.4 ± 0.1)^b^
	Crosslinked + 7d PBS	(56.0 ± 0.2):(44.0 ± 0.2)^b^
	Crosslinked + 14d PBS	(57.3 ± 0.1):(42.7 ± 0.1)^b^

^a^Determined by TGA analysis by applying Equation 1; ^b^Determined by gravimetric method by applying Equations 2 and 3.

### Mechanical Properties of the Bundles

**Figure 6:**
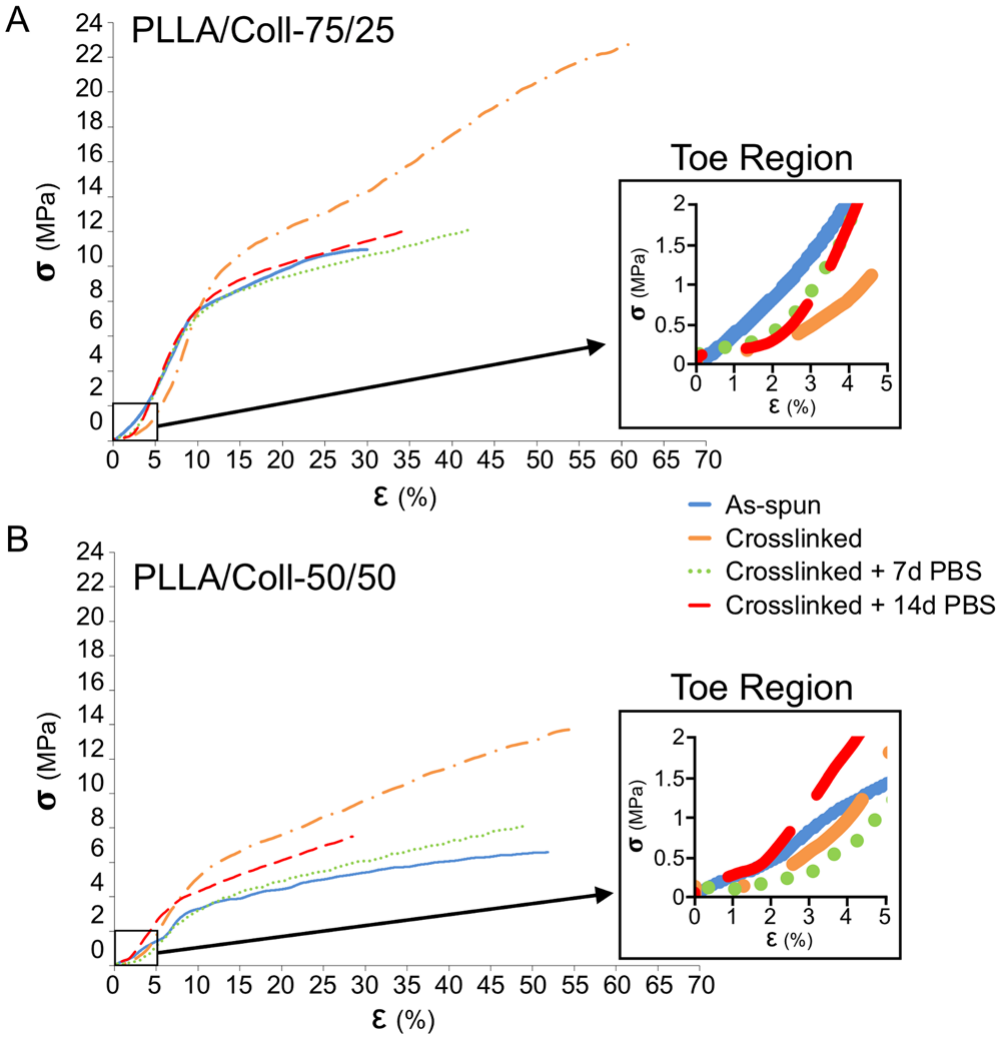
Representative stress-strain curves of the PLLA/Coll-75/25 (**A**) and PLLA/Coll-50/50 (**B**) bundles in four different conditions: as-spun, immediately after crosslinking (crosslinked), after crosslinking and immersion in PBS at 37 °C for 7 days (crosslinked + 7d PBS) and 14 days (crosslinked + 14d PBS). For both plots the initial toe region is also shown.

After hydration in PBS for 2 minutes, the stress–strain curves of the bundles as-spun for both blends showed a similar nonlinear behavior with an initial toe region, and a ductile behavior (Fig. 6B). Such ductility was maintained also after the crosslinking treatment and after ageing in PBS for 7 and 14 days.

After crosslinking, both blends showed a visible increase in mechanical properties, especially of the yield and failure stress. The mechanical properties progressively decreased after ageing in PBS with respect to the condition immediately after crosslinking, but even after 14 days they remained in the same range as the bundles as-spun (Fig. 6).

**Figure 7:**
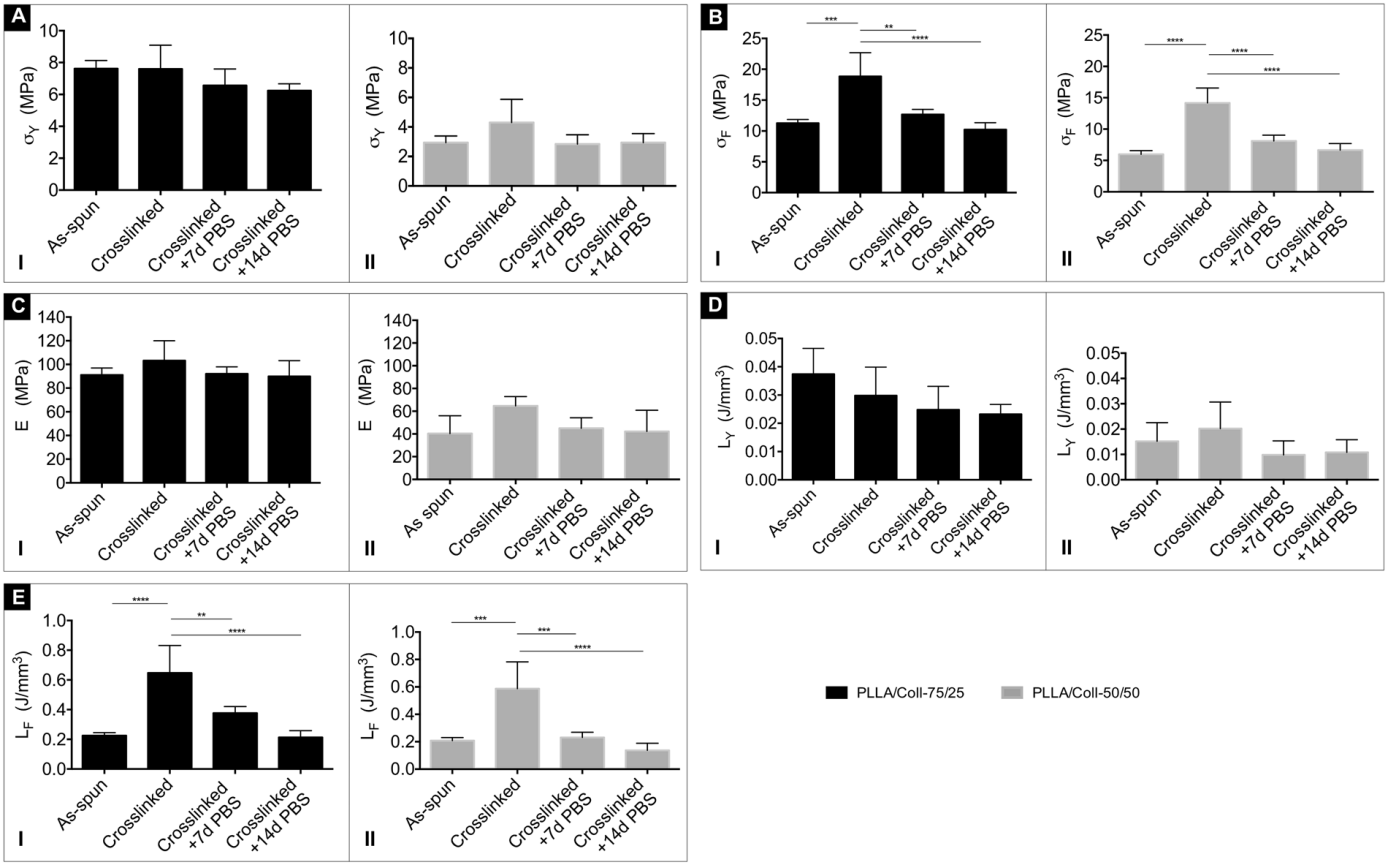
Mechanical properties of the bundles of PLLA/Coll-75/25 and PLLA/Coll-50/50 for the different conditions (as-spun, crosslinked, and after ageing in PBS). The following mechanical properties are reported: (**A**) yield stress (σ_Y_), (**B**) failure stress (σ_F_), (**C**) Young’s modulus, (**D**) work to yield (L_Y_), (**E**) work to failure (L_F_). The mean and standard deviation is plotted for the 5 samples tested for each condition. Statistical significance was assessed with a one-way analysis of variance (ANOVA), followed by post-hoc comparisons (Tukey multiple comparisons, *P ≤ 0.05, **P ≤ 0.01, ***P ≤ 0.001, ****P ≤ 0.0001).

The PLLA/Coll-75/25 bundles showed an increase in failure stress from 11.3 ± 0.6 MPa (as-spun) to 18.8 ± 3.8 MPa (immediately after crosslinking) and maintained a failure stress of 10.2 ± 1.1 MPa after 14 days in PBS. The PLLA/Coll-50/50 bundles showed an increase in failure stress from 6.0 ± 0.6 MPa (as-spun) to 14.2 ± 2.4 MPa (immediately after crosslinking) and after 14 days of ageing in PBS the failure stress was 6.6 ± 1.1 MPa. Moreover, as the crosslinking increased, the failure stress and failure strain also increased, and the crosslinked bundles displayed a higher work to failure. The PLLA/Coll-75/25 bundles had a work to failure of 0.225 ± 0.021 J/mm^3^ (as-spun), which increased to 0.647 ± 0.185 J/mm^3^ (immediately after crosslinking), and then decreased to 0.213 ± 0.045 J/mm^3^ after 14 days in PBS. For the PLLA/Coll-50/50 bundles the work to failure was 0.208 ± 0.022 J/mm^3^ (as-spun), and increased to 0.588 ± 0.195 J/mm^3^ (immediately after crosslinking) then decreasing to 0.137 ± 0.052 J/mm^3^ after 14 days in PBS. Some variations due to treatment were statistically significant (one-way ANOVA, Fig. 7). The highest yield and failure stress values were found for the PLLA/Coll-75/25 crosslinked bundles. In addition, the PLLA/Coll-75/25 showed higher values than the PLLA/Coll-50/50 also for the other mechanical properties (yield strain, Young’s modulus and work to yield) (Fig. 7). Conversely, the PLLA/Coll-50/50 had larger failure strain than the PLLA/Coll-75/25. Some differences between compositions were statistically significant (two-way ANOVA, Table S1).

### Cell Metabolic Activity and Morphology

**Figure 8:**
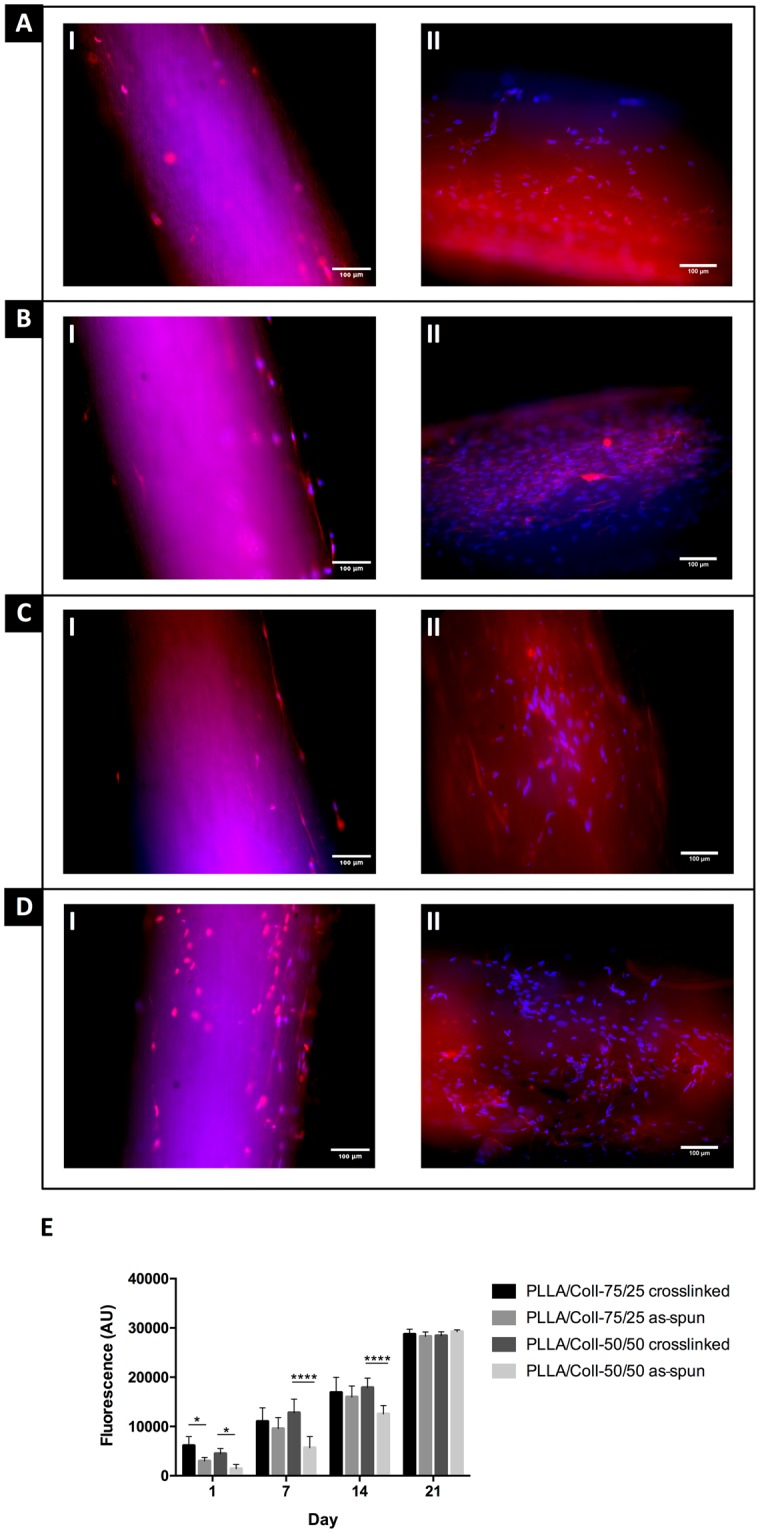
Fluorescence microscopy of NTF-322s with DAPI (cell nuclei, blue) and phalloidin-TRITC (actin, red) after (I) 14 days and (II) 21 days of NTF-322 culture on (**A**) PLLA/Coll-75/25 as-spun, (**B**) PLLA/Coll-75/25 crosslinked, (**C**) PLLA/Coll-50/50 as-spun and (**D**) PLLA/Coll-50/50 crosslinked bundles. The fibers exhibited some autofluorescence; however the cell nuclei could be clearly identified as discrete ellipses. (**E**) Comparison of NTF-322 cell metabolic activity assessed using a resazurin reduction assay after 1, 7, 14 and 21 days of culture. Initial attachment of the NTF-322s to the scaffolds was significantly lower in the scaffolds as-spun, compared to their crosslinked counterparts. Mean and standard deviation for 6 samples is reported. Statistical analysis was performed using two-way analysis of variance (ANOVA) followed by Tukey post-hoc test (*P ≤ 0.05, ****P ≤ 0.0001).

Cell attachment was higher on crosslinked bundles compared to non-crosslinked bundles of the same composition (Fig. 8). By day 7, cell metabolic activity, assessed by resazurin reduction, was similar in the PLLA/Coll-75/25 bundles (both as-spun and crosslinked) and in the PLLA/Coll-50/50 bundles crosslinked; only the PLLA/Coll-50/50 as-spun had significantly lower cell metabolic activity. By day 21, all bundle compositions supported similar cell metabolic activity. Only viable cells can exhibit metabolic activity, therefore this indicates that cells were viable on the bundles after 21 days of culture. The fluorescent images show that the cells were distributed over the length of the scaffolds and, after day 21, they were predominantly aligned with the nanofibers (Fig. 8A–D).

## Discussion

Tendon injuries still constitute an unsolved clinical need and are a major clinical problem for healthcare systems worldwide^2,3^. Recent advances in materials chemistry, biology and bioengineering have made 3D scaffolds available as a promising method to reinforce and replace tendons, providing structural support and a path for cells and new tissue formation. Synthetic scaffolds have been manufactured using a variety of polymers and fabrication methods from inert and resorbable polymers. In designing scaffolds for tendon tissue engineering, it is fundamental that the scaffold presents an appropriate 3D morphology and structure in order to mimic the complex hierarchical structures and mechanical properties of the tissue to replace^18–20^.

To face these challenges, in the present work bundles of axially aligned electrospun nanofibers were fabricated, with the aim of mimicking the fascicles of the human tendons. Among other fabrication methods, electrospinning appears to be an optimal technique, since aligned nano- and microfibrous mats, as well as fibrous bundles, can be produced with mechanical and morphological features similar to the tissue to be replaced. As a model material for the present study, blends of PLLA/Coll in two different compositions - PLLA/Coll-75/25 and PLLA/Coll-50/50 - were chosen, since it was previously shown that such materials allowed human-derived fibroblast adhesion and proliferation^17^. In order to prevent the collagen loss from the bundles, as well as to maintain the mechanical properties after ageing in PBS, in this paper a crosslinking treatment was carried out, by using the EDC/NHS reagents^42,43^. This chemical method has been largely employed in the literature to crosslink electrospun collagen scaffolds^28,44–46^. In particular, Barnes *et al*. have crosslinked electrospun collagen with EDC by immersion in ethanol instead of aqueous solution during the crosslinking treatment, to better preserve the fibrous morphology^46,47^. EDC enables activation of the carboxylic acids of the aspartic and glutamic acid residues present along the chains of collagen, which subsequently react with the amine functions of lysine residues of other chains. To maximize the effectiveness of EDC, it is also necessary to introduce NHS that converts the O-acylisourea group of EDC into an NHS-activated carboxylic acid group, which is reactive towards amine groups of lysine^48^. At the end of the reaction, EDC is not linked between the collagen residues, but it leaves the process as a 1-ethyl-3-(3-dimethyl- aminopropyl) urea by-product. Commonly, EDC and NHS are used in water based solutions, but Barnes *et al*. showed that when ethanol is used the fibrous morphology is better preserved^42,46,47,49,50^. In the present work measures of weight loss demonstrated that this collagen crosslinking procedure successfully maintained the collagen component in the bundles, since uncrosslinked collagen chains are expected to quickly dissolve upon water contact. It can be noted that the crosslinking procedure is less effective on PLLA/Coll-75/25 compared to PLLA/Coll-50/50 (the former lost 30% of collagen while the latter only 15%), probably due to the increased number of PLLA chains leading to a reduced amount of collagen crosslinking sites.

In order to evaluate the 3D-structure and morphology of the produced scaffolds and to make comparison with the architecture of the native tendon fascicles, a thorough morphological analysis was performed by means of SEM and XCT^18–20,51^. These imaging techniques were also useful to assess the effect of the crosslinking treatment and of the immersion in PBS for 7 and 14 days on the morphology of the nanofibers. Interestingly, the mean diameters of the nanofibers of both blends (in the range 0.35–0.40 μm, measured by SEM) were in line with the range of the fibrils in the human tendons, such as in the Achilles^52^. The crosslinking process did not modify the average diameter and the morphology of the nanofibers. The nanofibers also remained in the same dimensional range even after 7 days in PBS, whereas a slight increase in fiber diameter was observed after 14 days in PBS.

The manufacturing process proposed in this paper enabled highly aligned electrospun nanofibers arranged in bundles with length of several centimeters to be fabricated. The versatility of the proposed fabrication workflow allows the diameter of the bundles to be tailored to the required structure by adjusting the mat thickness and the wrapping process. Bundles of both PLLA/Coll blends were produced with a diameter in the same range of tendon fascicles, such as Achilles, reported in the literature, even after the slight collagen loss occurring during the crosslinking process that caused a slight narrowing of the bundles^18–20^.

While SEM analysis allowed investigation of the morphology of the nanofibers on the outer surface of the bundles, XCT analysis was used to evaluate detailed morphology of fiber distribution within the bundles at submicron resolution. The XCT analysis confirmed that the internal morphology and alignment of the nanofibers were in agreement with those obtained through SEM on the surface of the bundles. It was found that, while the nanofibers were mainly aligned within the bundles, a fraction of nanofibers exhibited a range of alignments (Figs 3–5): this represents a desirable feature to mimic the morphology of the human tendon^18–20^. This morphology was maintained also after the crosslinking process. Producing XCT images with such voxel size is critical to minimize partial volume effects, but is very challenging. Only a few studies so far were able to produce sub-micron imaging of electrospun nanofibers. Farrugia *et al*. produced high-resolution tomographic images of poly(ε-caprolactone) (PCL) electrospun microfibers (mean fiber diameter 7.5 ± 1.6 μm) with a voxel size of 0.79 μm^53^. Later Bosworth *et al*. imaged PCL electrospun nanofibers yarns (mean diameter 0.4 μm) with a resolution of 0.61 μm^29,31^. Kogikoski *et al*. obtained XCT images of PCL/Polyaniline blends electrospun nanofibers (mean diameter 0.6–0.3 μm) with a voxel size of 3.37 μm^54^. Finally, Bradley *et al*. performed XCT on electrospun mats of poly(lactide-co-glicolide) (PLGA) microfibers (mean diameter 4.0 μm) with a voxel size of 0.13 μm^55^. However, to the best of our knowledge we were the first group to obtain high quality XCT images of electrospun PLLA/Coll nanofibers and we were able to have a complete characterization of the bundles’ nanostructure. In fact, obtaining XCT images of such bundles and nanofibers is very challenging because: (i) it is very difficult to resolve the collagen due to its low radiographic density, without using contrast agents; (ii) the mean diameter of the nanofibers was close to the pixel size resolution of the scan^56^.

The mechanical tests (Fig. 6) confirmed that both blends in all the tested conditions had an initial highly compliant toe region similar to that of the natural tendon fascicles^57^. The two bundles’ compositions, as-spun and after crosslinking and ageing in PBS for 7 and 14 days, showed a ductile behavior (Fig. 6). As expected, hydration in PBS before the test increased the ductility of the bundles of both compositions, compared to dry samples^17^. The crosslinking process significantly improved the mechanical performance (especially the failure stress and work to failure) of the bundles of both compositions (Fig. 7). In addition, crosslinking preserved the mechanical properties after ageing compared to the non-crosslinked version^17^. The ductile behavior of the hydrated bundles guarantees a safety factor in case of overload, which is an essential requirement for a strenuously loaded orthopedic device.

The mechanical tests indicate that the PLLA/Coll-75/25 bundles are superior to the PLLA/Coll-50/50 in terms of yield stress, failure stress and of Young’s modulus (Fig. 7) (all values of the bundles’ mechanical properties, alongside the values for human tendon fascicles^57^, are listed with mean and standard deviation in Table S2). Furthermore, the failure stress of the PLLA/Coll-75/25 bundles (also after 14 days of ageing in PBS) was slightly lower, but in the same range as the fascicles of different human tendon (range: 6.8–28.1 MPa, Table S2)^57^. It must be noted that while the fascicles of the natural tendon exhibits a rather sudden failure (at a higher stress than the bundles of the present study), the bundles exhibit a pronounced post-yield region. The different mechanical properties of the two tested compositions can be ascribable to the specific contribution provided by each component: after crosslinking, electrospun collagen behaves as a rigid and fragile material^46^ while the PLLA component, being more ductile, provides higher elongation and plasticity. The fact that the artificial scaffold is weaker than the natural tendon is desirable in terms of patient safety: in fact, to avoid damage in the patient’s repaired site in case of overload, failure should start in the implanted device, rather than in the host tissue.

The Young’s modulus of both blends was slightly lower than that of the Achilles tendon fascicles (i.e. 222.8 ± 84.6 MPa (Afro-American) and 316.8 ± 110.0 (Caucasian)), but in the same range of iliopsoas tendon fascicles (i.e. 165.3 ± 67.3 MPa (Afro-American) and 63.5 ± 23.6 (Caucasian)) reported in literature^17,57^. However, this discrepancy might not be critical since these kinds of resorbable electrospun scaffolds are meant to serve as a temporary replacement and allow tenocyte proliferation while the limb is only partially loaded to prevent damage. Progressive replacement of the electrospun nanofibers with the physiological triple helix collagen expects to progressively increase the stiffness of the regenerated tendon.

Comparisons with previous studies are difficult, as no other research groups have investigated the mechanical properties of electrospun bundles of PLLA/Coll blends, apart from our previous work^17^. Among others, some works reported the mechanical properties of bundles and yarns for tendon tissue regeneration. Bosworth *et al*. tested nanofibrous yarns of poly(ε-capro-lactone) (PCL) finding a Young’s modulus of 14.11 ± 3.76 MPa and a failure stress of 4.74 ± 1.64 MPa^31^. Pauly *et al*. tested bundles of aligned nanofibers of PCL with a Young’s modulus of about 40 MPa and a failure stress of about 14 MPa^33^. Domingues *et al*. investigated the mechanical properties of aligned bundles of nanofibers, made of PCL/Chitosan/Cellulose nanocrystals, finding a Young’s modulus of 540.5 ± 83.7 MPa and a failure stress of 39.3 ± 1.9 MPa^58^.

Biological evaluation showed that both bundle compositions supported cell attachment and growth both crosslinked and as-spun. Crosslinking collagen via EDC has been previously shown by Haugh *et al*. to reduce cellular attachment when used at high concentrations (>6 mM)^59^. This was theorized to happen due to the cytotoxic effects of urea - a by-product formed by the crosslinking reaction that remained trapped within the scaffold at higher EDC concentrations, which is in contrast to the increased attachment found here. However, it should also be noted that the study by Haugh *et al*. used collagen-glycosaminoglycan scaffolds and the increased cellular attachment found here could be due to the retention of the Coll within the bundles due to the crosslinking, as previously shown^17,59^. Other studies have also shown that improved Coll retention in the PLLA/Coll bundles improved attachment and cytocompatibility in comparison to unmodified PLLA^60^. The improved attachment can be attributed to increased hydrophilicity of the PLLA/Coll in comparison to unmodified PLLA and to the introduction of bioactive factors^60,61^, that are maintained in the natural polymeric chains, although the triple helix collagen structure is lost after electrospinning, as extensively demonstrated in other works^46,62,63^.

This study focused on the suitability of the mechanical properties, morphology and cell metabolic activity of the bundles to be used as a scaffold for tendon regeneration. With respect to our previous work^17^ this study demonstrates that electrospun bundle of aligned fibers with a proper and controlled collagen content and tested under physiological conditions possess unprecedented stable mechanical and biological properties. In future it would be important to address a possible means of attachment of the scaffold at the extremities, either to the residual tendon, or to the bone insertion, or to the muscle extremity.

## Conclusion

This study proposed a technique for fabricating electrospun bundles made of PLLA/Collagen blends for tendon repair. The analysis of the directionality of the fibers obtained via high-resolution x-ray computed tomography (XCT) indicated a satisfactory alignment and the scatter of the nanofibers, mimicking natural tendon. The mechanical properties of the bundles after crosslinking were comparable to those required to replace/regenerate tendinous tissue, and were well-preserved even after ageing in PBS. The most promising composition in terms of mechanical properties was the PLLA/Coll-75/25 blend. Finally, the crosslinked bundles supported good cell metabolic activity when seeded with fibroblasts. While this study focused on the use of PLLA/Coll electrospun scaffolds for tendon regeneration, possible future applications could include repair of the ligaments, which have similar composition and microstructural arrangement of the collagen nanofibers in the tendon.

## Experimental Section

### Materials

Acid soluble collagen type I (Coll), extracted from bovine skin (Kensey Nash Corporation d/b/a DSM Biomedical, Exton, USA) and Poly(L-lactic acid) (PLLA) (Lacea H.100-E, M_w_ = 8.4 × 10^4^ g mol^−1^, PDI = 1.7, Mitsui Fine Chemicals, Dusseldorf, Germany) were used. 2,2,2-Trifluoroethanol (TFE), 1,1,1,3,3,3-Hexafluoro-2-propanol (HFIP), Dichloromethane (DCM), Dimethylformamide (DMF), N-(3-Dimethylaminopropyl)-N′-ethylcarbodiimide hydrochloride (EDC) and N-Hydroxysuccinimide (NHS) (Sigma-Aldrich, Staint Louis, USA) were used as received. The following polymeric solutions were used: (i) PLLA/Coll-75/25 (w/w) prepared from a 15% (w/v) solution of PLLA and Coll dissolved in TFE:HFIP = 50:50 (v/v) (1.125 g of PLLA and 0.375 g of Coll were dissolved in 10 mL); (ii) PLLA/Coll-50/50 (w/w) prepared from a 15% (w/v) solution of PLLA and Coll dissolved in TFE:HFIP = 50:50 (v/v) (0.75 g of PLLA and 0.75 g of Coll were dissolved in 10 mL).

### Electrospinning

Bundles were fabricated using a laboratory electrospinning machine (Spinbow Lab Unit, Spinbow S.r.1., Bologna, Italy), equipped with a linear sliding spinneret (carrying two syringes ejecting the same polymer solution) and a rotating drum collector (diameter = 150 mm; length = 500 mm) (Fig. S1). To electrospin each of the solutions, a syringe pump (KD Scientific 200 series, Hillinois, USA) and two glass syringes containing the same polymer solution and connected to two stainless-steel blunt-ended needles (inner diameter = 0.51 mm) with PTFE tubes, were used. Electrospinning was performed at room temperature (RT) and relative humidity 20–30%. Both blends were electrospun in the following conditions: applied voltage = 22 kV, feed rate = 0.5 mL h^−1^, electrospinning time = 2 hours. A high-speed rotating aluminum drum collector (peripheral speed = 22.8 m s^−1^), positioned 200 mm away from the needle tips, was used to produce mats made of nanofibers preferentially aligned in the direction of drum rotation. The sliding spinneret with the two needles had an excursion of 120 mm, with a sliding speed of 1200 mm min^−1^. The mats made of aligned nanofibers were cut circumferentially into strips, and manually wrapped to produce the bundles (Fig. 1). By fixing the time of electrospinning and the width of the strips to be wrapped, bundles of approximately 550–650 μm in diameter were obtained. Thus, the final bundles were as long as the circumference of the rotating drum collector (i.e. about 470 mm), and were made of axially aligned nanofibers (Fig. 1B).

### Crosslinking Treatment

Bundles of PLLA/Coll-75/25 and PLLA/Coll-50/50 were immersed for 24 hours at RT under mild agitation in a crosslinking solution of EDC and NHS 0.02 M in 95% ethanol, adapted from a previously reported procedure^64^. The samples were then immersed in phosphate buffer saline (PBS, 0.1 M, pH = 7.4) for 30 min, thoroughly washed in distilled water for 2 hours (by changing water every 15 min) and dried over P_2_O_5_ under vacuum at RT.

### Imaging and Morphological Analysis

Scanning Electron Microscopy (SEM) (Philips 515 SEM, Amsterdam, Netherlands) observations were carried out using an accelerating voltage of 15 kV, on samples sputter-coated with gold. The distribution of nanofiber diameters was measured on the SEM images of approximately 200 nanofibers, by using the software ImageJ^65^. The two-way ANOVA followed by the Tukey post-hoc test was used to test the statistical significance of the differences between means.

High-resolution images of both compositions as-spun and immediately after crosslinking were acquired with a high-resolution x-ray computed tomography (XCT, Xradia Versa 510, ZEISS, Pleasanton, CA, USA), with two different isotropic voxel sizes. (i) Images with 1 μm voxel size were collected at rotational steps of 0.18 over 360° (scanning time: 6 h); (ii) Images with 0.4 μm voxel size were collected at rotational steps of 0.18 over 360° (scanning time: 10 h). For both voxel sizes the same settings were used: 40 kV Voltage, 3 W Power, 75.5 μA tube current. The images were reconstructed using ZEISS Scout-and-Scan Reconstructor software and were visualized using the XM3DViewer1.2.8 software. To measure the nanofiber orientation, the scans at 0.4 μm voxel size were analyzed with ImageJ: first the scans were resliced for an axial view of the nanofibers, then the Directionality plugin of ImageJ was used, which exploits the Local Gradients orientation method^65–67^.

### Instrumental Characterization

Thermogravimetric analysis (TGA) was performed (TGAQ500 analyzer, TA Instruments, New Castle, USA) from RT to 700 °C (heating rate 10 °C min^−1^, nitrogen gas). TGA was performed on the as-spun bundles of both blends, and on pure PLLA and collagen powders. Since pure collagen shows a considerable residual weight at 700 °C while PLLA residual weight is very low, it can be assumed that the weight residues of the bundles are proportional to the amount of collagen. Therefore, by comparing the residual weights at 700 °C, the real composition of the bundles was determined by applying the following linear system:

1 $$\begin{array}{c}{Wt}{ \% }_{{res}}^{{Coll}}\cdot {x}+{Wt}{ \% }_{{res}}^{{PLLA}}\cdot {y}={Wt}{ \% }_{res}^{{Blend}}\\ x+y=1\end{array}$$

where ${Wt}{ \% }_{{res}}^{{Coll}}$ is the residual weight percentage of pure collagen (26.7%); ${Wt}{ \% }_{{res}}^{{PLLA}}$ is the residual weight percentage of pure PLLA (1.6%); ${Wt}{ \% }_{{res}}^{{Blend}}$ is the residual weight percentage of PLLA/Coll bundles (7.8% for PLLA/Coll-75/25 and 14.9% for PLLA/Coll-50/50); x and y are the weight fractions of Collagen and PLLA in the bundles, respectively.

### Collagen Loss from the Crosslinked Bundles

In order to determine collagen loss after the crosslinking treatment and after immersion in PBS for 7 and 14 days at 37 °C after the crosslinking treatment, triplicate samples of as-spun bundles (about 40 mg each) were dried over P_2_O_5_ under vacuum at RT and weighed to obtain the initial mass. The samples were crosslinked, washed and dried, as described above. The samples were individually immersed in 3 mL of PBS with sodium azide (Sigma-Aldrich, Saint Louis, USA) and incubated in a water bath (SW22, Julabo, Milan, Italy) at 37 °C with shaking at 80 rpm. At 7 and 14 days, the samples were recovered from the bath, gently washed with distilled water for 2 and a half hours, dried over P_2_O_5_ under vacuum and weighed. The weight loss was entirely ascribed to the dissolution of non-crosslinked collagen. This assumption is supported by the fact that PLLA mats incubated in PBS do not show any weight loss in the time range investigated (up to 14 days)^17^.

The bundle composition in terms of weight content of PLLA (wt%_PLLA_) and of Collagen (wt%_Coll_) after crosslinking, immersion in PBS for 7 days and immersion in PBS for 14 days, was calculated by applying Equations 2 and 3:

2 $${\mathrm{wt} \% }_{{\rm{PLLA}}}=\frac{{{\rm{m}}}_{{\rm{in}}}\cdot {{\rm{w}}}_{{\rm{PLLA}}}}{{{\rm{m}}}_{{\rm{fin}}}}.100$$

3 $${\mathrm{wt} \% }_{{\rm{Coll}}}=100-{\mathrm{wt} \% }_{{\rm{PLLA}}}$$

where m_in_ is the initial dry weight, m_fin_ is the dry weight after treatment and w_PLLA_ is the initial PLLA weight fraction (i.e. w_PLLA_ = 0.75 for PLLA/Coll-75/25 and w_PLLA_ = 0.5 for PLLA/Coll-50/50).

### Mechanical Characterization of the Bundles

In order to evaluate the mechanical properties, also in relation to collagen crosslinking and to ageing in PBS, destructive tensile tests were performed on bundles of both blends as-spun, after crosslinking, and on the crosslinked bundles aged in PBS for 7 and 14 days. To measure the diameter of each bundle, a light optical microscope (Axioskop, ZEISS, Oberkochen, Germany) equipped with a camera (AxioCam MRc, ZEISS, Oberkochen, Germany) was used (mean and standard deviation of 10 measurements). The section was measured on dried specimens, immediately after preparation of the bundles, and also after crosslinking and immersion in PBS. The samples were immersed in PBS for two minutes before the tensile test. The mechanical tests (5 samples per treatment group) were carried out with a servo-hydraulic testing machine (8032, Instron, High Wycombe, UK), with a ±1kN dynamic cell (Instron, precision class 0.5, High Wycombe, UK). Selection of the appropriate range and signal filtering allowed measuring the force with a precision of 0.02 N. Dedicated capstan grips (Fig. S2) were used to limit the stress concentrations at the extremities. The gauge length was 47.42 mm (consistent with BS EN 12562:1999 and the ASTM D2256/D2256M-10(2015) Standards, this included the free length and the portion of specimen wrapped around the capstans). The test machine was operated in displacement control, with an actuator speed of 16 mm.s^-1^ (resulting in a strain rate of 33% s^−1^: this is in the range of strain rates experienced by the tendon during a variety of physiological tasks^68,69^). The load-displacement curves were converted to stress-strain curves using the cross-section area measured on dry samples. The following indicators were extracted: yield stress (σ_Y_), yield strain (ε_Y_), failure stress (σ_F_), failure strain (ε_F_), Young’s modulus (E), work to yield (L_Y_), work to failure (L_F_). The significance of the effect of the crosslinking and the ageing in PBS on the two blends was assessed with two-way ANOVA followed by Tukey post-hoc, while the effect of the crosslinking and the ageing in PBS on the same composition was assessed with one-way ANOVA, followed by the Tukey post-hoc.

### Biological Evaluation

Non-tumoral human fibroblasts (NTFs), obtained from waste tissue collected under ethical approval 09/H1308/66 from the NRES Committee Yorkshire and The Humber, Sheffield (Informed consent was provided for the collection and use of surgical waste tissue for research), were cultured in basal medium (BM) consisting of α-MEM culture medium (Lonza^®^, Slough, UK), 10% foetal bovine serum (FBS, Labtech, Heathfield, UK), 2 mM L-glutamine (Sigma Aldrich, Saint Louis, USA) and 100 mg mL^−1^ penicillin/streptomycin (Sigma Aldrich, Saint Louis, UK). NTFs were cultured in 75 cm^2^ tissue-culture flasks at 37 °C in 5% CO_2_ in a humidified atmosphere with media changes every 2–3 days. Cells were used between passage 4 and 6. The electrospun bundles were cut to 1 cm in length and sterilized by soaking in 70 vol% ethanol for 1 h before being washed 3 times in PBS (Sigma Aldrich, Saint Louis, USA). The bundles were seeded with 50,000 cells at a density of 1,000,000 cells mL^−1^ in a 24 well plate. The cells were left for 45 minutes to attach, after which 1 mL of BM was added to each well to submerge the bundles. Resazurin reduction (RR) assay was used to measure the metabolic activity of the cells attached to the bundles. A RR was performed at 4 time points (day 1, 7, 14 and 21). Before each RR the bundles were transferred into a new 24 well plate to ensure the metabolic activity of only the cells attached to the bundles was measured. 1 mL of 0.1 mM resazurin salt solution in BM was added to each well and incubated in the dark for 4 hours at 37 °C. During this period, the non-fluorescent blue resazurin solution is reduced by the cells to resorufin, a highly fluorescent pink solution. 200 µL of the reduced solution was transferred to a 96 well plate and measured using a spectrofluorometer (FLX800, BIO-TEK Instruments Inc., Winooski, USA) at an excitation wavelength of 540 nm and an emission wavelength of 630 nm. The bundles were washed twice with PBS before fresh BM was added.

To assess the distribution of cells on the bundles, the samples were stained for cell nuclei on day 7 of culture. The bundles were fixed in 3.7% formaldehyde (Sigma Aldrich, Saint Louis, USA) for 15 minutes before permeabilization with 0.1% v/v Triton X-100 (Sigma Aldrich, Saint Louis, USA) in PBS for 10 minutes. 5 uM phalloidin-TRITC (Sigma Aldrich, Saint Louis, USA) in PBS was applied for 30 minutes to stain actin followed by 3 PBS washes. 1 µg/mL 4′-6-diamidino-2-phenylindole (DAPI, Sigma Aldrich, Saint Louis, USA) was applied for 15 minutes to stain the nuclei. The samples were visualized with a microscope (Eclipse Ti, Nikon, Tokyo, Japan) equipped with a camera (Intensilight C-HGFI, Nikon, Tokyo, Japan). Cell metabolic activity was compared using a two-way analysis of variance (ANOVA) followed by the Tukey post-hoc test.

## Electronic supplementary material

Supporting Information

Movie of PLLA/Coll-75/25 as-spun bundle

Movie of PLLA/Coll-75/25 crosslinked bundle

## Data Availability

All data generated or analyzed during this study are included in this published article (and its Supplementary Information Files).
