# Supplementary material for: Tendon Fascicle-Inspired Nanofibrous Scaffold of Polylactic acid/Collagen with Enhanced 3D-Structure and Biomechanical Properties
Source: Sci Rep. 2018 Nov 21;8:17167. doi: 10.1038/s41598-018-35536-8 (PMC6249227; doi:10.1038/s41598-018-35536-8)
Supplement: Supplementary file 1 — Supporting Information [file 41598_2018_35536_MOESM1_ESM.docx]

Supporting Information

Tendon Fascicle-Inspired Nanofibrous Scaffold of Polylactic acid/Collagen with Enhanced 3D-Structure and Biomechanical Properties

Alberto Sensini, Chiara Gualandi, Luca Cristofolini, Andrea Zucchelli, Gwendolen C. Reilly, Liam Boyle, Alexander P. Kao, Gianluca Tozzi, Maria Letizia Focarete*


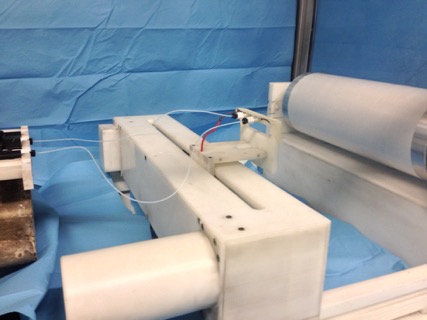


**Figure S1.** Electrospinning setup. The pump-driven syringes are visible on the left. The two needles were mounted on a motorized sliding spinneret (at the center of the picture) and were connected to a positive high voltage. The high-speed rotating drum collector (visible on the right) was connected to the ground.

**Figure S2.** Capstan grips designed to perform the tensile test on the bundles (A, scale bar = 10 mm). Typical stress-strain plot (B): the initial toe region was disregarded; the failure stress (V) was identified as the highest stress in the entire curve; the starting point of the linear region (I) was univocally identified as 20% of failure stress; an initial guess for the yield strain was visually identified (III); the initial linear regression (solid line) was applied to the first 50% of the linear region, between points I and II (which was half-way between I and III); a second line parallel to the initial regression was drawn, with an offset of 0.5% strain (dashed line); the limit of proportionality was defined with the 0.5%-strain offset criterion as the intersection (IV) between the latter line and the stress-strain curve; the Young modulus was calculated as the slope of a new regression line between I and IV. The work to yield and to failure were calculated as the integrals under the curves (with the method of trapezoids).


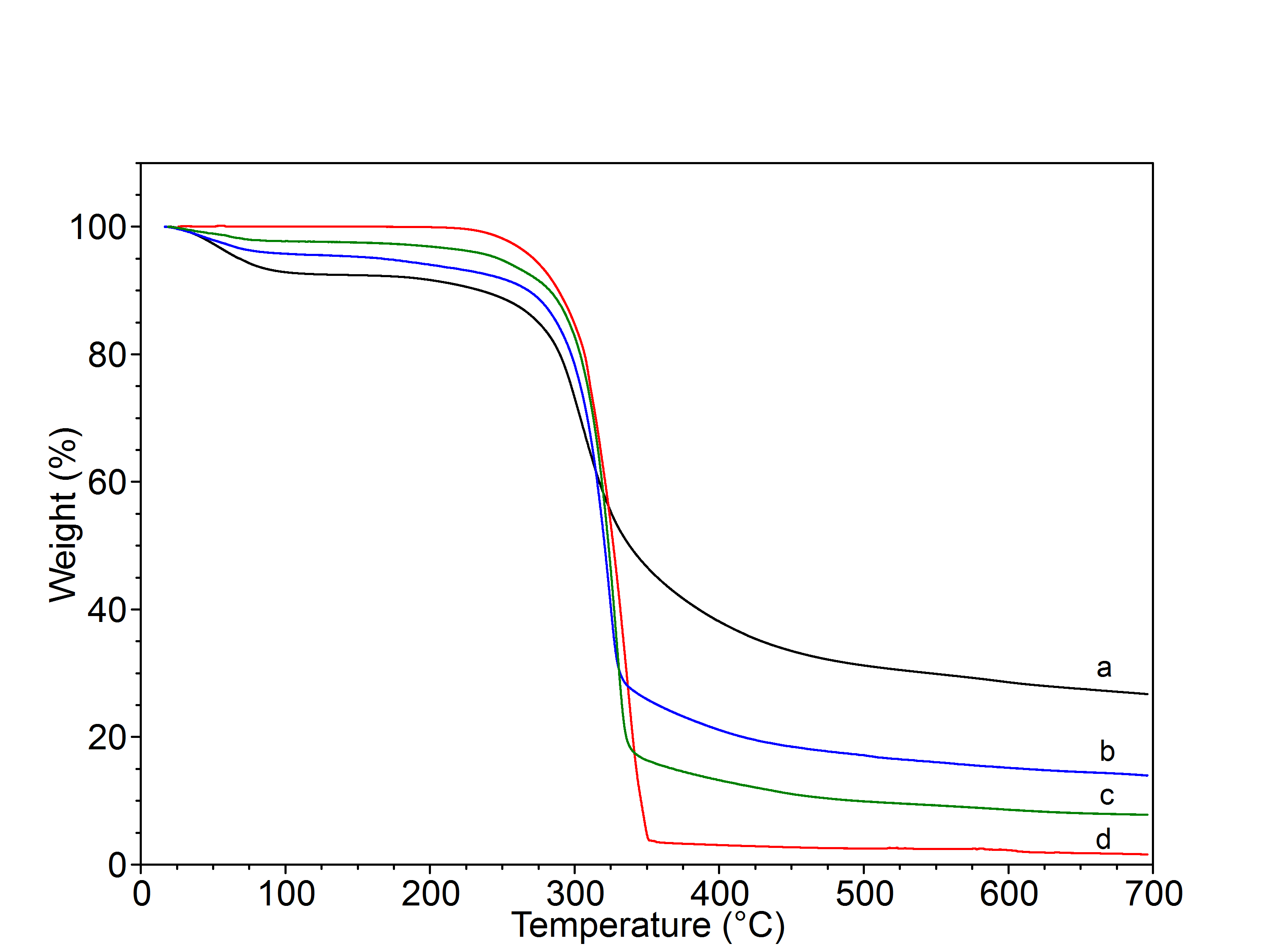


**Figure S3.** TGA analysis of collagen powder (a), of the as-spun bundles of PLLA/Coll-50/50 (b), and PLLA/Coll-75/25 (c) and of PLLA pellets (d).

**Table S1.** Comparison of mechanical properties of PLLA/Coll-75/25 and PLLA/Coll-50/50 exposed to the same treatment (as-spun, crosslinking) and ageing in PBS for the same periods, using two way ANOVA. The mean and standard deviation are plotted for the two compositions, together with statistical significance of post-hoc comparisons (Tukey multiple comparisons, * P≤ 0.05, ** P≤ 0.01, *** P≤ 0.001, **** P≤ 0.0001, ns = not significant).

|  | Yield stress | Failure stress | Yield strain | Failure strain | Young modulus | Work to yield | Work to failure |
| --- | --- | --- | --- | --- | --- | --- | --- |
| As-spun | **** | ** | ns | ** | *** | ** | ns |
| Crosslinked | ** | ** | ns | ns | ** | ns | ns |
| Crosslinked + 7d PBS | *** | ** | ns | ns | *** | ns | ns |
| Crosslinked + 14d PBS | *** | * | ns | ns | *** | ns | ns |

**Table S2.** Mechanical properties of PLLA/Coll-75/25 and PLLA/Coll-50/50 exposed to the same treatment (as-spun, crosslinking) and ageing in PBS for the same periods, and the mechanical properties of human tendon fascicles reported by Hanson et al. (i.e. AA=Afro-American man; CC=Caucasian man)^1^ The mean and standard deviation are plotted for each value.

| **PLLA/Coll-75/25** | Yield stress  (MPa) | Failure stress  (MPa) | Yield strain  (%) | Failure strain  (%) | Young modulus  (MPa) | Work to yield  (J/mm^3^) | Work to failure  (J/mm^3^) |
| --- | --- | --- | --- | --- | --- | --- | --- |
| As-spun | 7.6±0.5 | 11.3±0.6 | 10.5±1.8 | 29.6±2.3 | 91.1±5.9 | 0.038±0.0092 | 0.225±0.021 |
| Crosslinked | 7.6±1.5 | 18.8±3.8 | 11.2±1.6 | 54.6±5.5 | 103.2±16.8 | 0.030±0.010 | 0.647±0.185 |
| Crosslinked + 7d PBS | 6.6±1.0 | 12.7±0.8 | 9.9±1.3 | 44.6±4.8 | 92.0±5.9 | 0.025±0.0081 | 0.377±0.044 |
| Crosslinked + 14d PBS | 6.2±0.4 | 10.2±1.1 | 11.5±2.2 | 32.0±4.9 | 89.9±13.3 | 0.023±0.0036 | 0.213±0.045 |
| **PLLA/Coll-50/50** |  |  |  |  |  |  |  |
| As-spun | 2.9±0.4 | 6.0±0.6 | 9.8±1.9 | 50.1±1.7 | 40.4±15.6 | 0.015±0.0072 | 0.208±0.022 |
| Crosslinked | 4.8±1.2 | 14.2±2.4 | 9.1±2.2 | 63.8±12.5 | 64.7±8.1 | 0.020±0.011 | 0.588±0.195 |
| Crosslinked + 7d PBS | 2.8±0.6 | 8.1±0.9 | 8.8±3.4 | 46.7±5.4 | 45.0±9.2 | 0.010±0.0055 | 0.231±0.038 |
| Crosslinked + 14d PBS | 3.0±0.6 | 6.6±1.1 | 8.9±2.5 | 34.4±12.8 | 42.1±18.7 | 0.011±0.0049 | 0.137±0.052 |
| **Human collagen fascicles**^1^ |  |  |  |  |  |  |  |
| Achilles’ tendon (AA) | - | 21.9±9.9 | - | 16.3±3.5 | 222.8±84.6 | - | - |
| Achilles’ tendon (CC) | - | 28.1±9.8 | - | 13.8±4.4 | 316.8±110.0 | - | - |
| Iliopsoas tendon (AA) | - | 22.5±7.3 | - | 19.7±5.2 | 165.3±67.3 | - | - |
| Iliopsoas tendon (CC) | - | 6.8±2.1 | - | 18.3±3.5 | 63.5±23.6 | - | - |

**References**

**1.** Hanson, P., Aagaard, P. & Magnusson, S. P. Biomechanical properties of isolated fascicles of the Iliopsoas and Achilles tendons in African American and Caucasian men. Ann. Anat. 194, 457–460 (2012)

**Movies**

**75_25_as_spun.mov.** XCT movie at 0.4 μm voxel size of a PLLA/Coll-75/25 as-spun bundle.

**75_25_crosslinked.mov.** XCT movie at 0.4 μm voxel size of a PLLA/Coll-75/25 crosslinked bundle.
